# Phenylalanine intercalation parameters for liquid-disordered phase domains – a membrane model study

**DOI:** 10.1186/s13628-018-0047-z

**Published:** 2018-11-15

**Authors:** Paulina Adamczewski, Valeria Tsoukanova

**Affiliations:** Department of Chemistry, York University, Toronto, ON M3J 1P3 Canada

**Keywords:** Phenylalanine, Langmuir monolayer, Epifluorescence microscopy, Amino acid/phospholipid interaction, Amyloid fibrils

## Abstract

**Background:**

Propensity of phenylalanine (Phe) for nonpolar environments drives its intercalation into phospholipid membranes, which has been implicated in metabolic and neurological disorders. The knowledge of Phe intercalation parameters can be instrumental in understanding various membrane processes triggered by interactions with Phe, in particular the early events leading to the formation of nucleation/docking sites for the self-assembly of Phe amyloid fibrils at the membrane surface.

**Results:**

In this study, we used monolayers of phosphatidylethanolamine (DPPE) and phosphatidylcholine (DPPC) to mimic the membrane outer leaflet. Its initial interaction with Phe was modeled by injecting Phe into the aqueous phase underneath the monolayer. Constant pressure insertion assays augmented with epifluorescence microscopy imaging were used to monitor Phe intercalation. Our primary goal was to determine the Phe intercalation area, *A*_Phe_. Two values were obtained for *A*_Phe_, 33 ± 2 and 48 ± 3 Å^2^.

**Conclusions:**

Phe appeared to discriminate between DPPE and DPPC packing, and use two modes of intercalation. The area of *A*_Phe_ 33 ± 2 Å^2^ is consistent with a Phe monomer intercalating into membrane by inserting the phenyl ring nearly normal to the membrane surface. This mode has been found to operate in DPPE membranes. For DPPC membranes however, the value of *A*_Phe_ = 48 ± 3 Å^2^ suggests that, from saline, Phe tends to intercalate as a larger species plausibly dragging along a counterion, Na^+^, in a Na^+^(Phe) complex.

**Electronic supplementary material:**

The online version of this article (10.1186/s13628-018-0047-z) contains supplementary material, which is available to authorized users.

## Background

Phenylalanine (Phe) is an amino acid with a nonpolar aromatic side chain. Phe has one of the highest hydrophobicity scales, which drives it into nonpolar environments, e.g. the hydrocarbon core of phospholipid membranes [1–6]. On one hand, this offers a great therapeutic potential. The incorporation of Phe residues has indeed demonstrated to drastically increase the potency of antimicrobial peptides plausibly by enabling stronger interaction with the bacterial cell envelope [7, 8]. On the other hand, the propensity of Phe for the membrane hydrocarbon core together with its tendency to cluster has been implicated in metabolic and neurological disorders [6, 9–14]. Phe residues have been identified as major amyloidogenic sites in proteins and peptides involved with proteopathies, e.g. Alzheimer’s and prion diseases, type II diabetes, etc. [14]. Phe is also the only amino acid capable of self–assembling into supramolecular structures with amyloid morphology [6, 9–13]. Interactions of these structures with membrane phospholipids have been suggested to cause cell toxicity, in particular in phenylketonuria, by perturbing the phospholipid packing and compromising the membrane structural integrity [9, 10]. This has thus put Phe/membrane interactions into the focus of renewed research interest over the recent years [5, 6, 12, 13, 15, 16].

A number of studies have examined Phe self-assembly and interactions with model phospholipid membranes [1, 5, 6, 9–13, 15–17]. It has been reported that Phe is capable of intercalating into the membrane through small-scale packing defects [1, 5, 15], forming Phe/phospholipid complexes [15–17], self-assembling into tubular pore- and needle-like structures piercing through the membrane [6, 11, 13], and depositing fibrils onto the membrane surface [9, 12]. The depth of Phe localization in the membrane has been found to vary significantly, from lying nearly flat at the membrane surface to being embedded into the hydrocarbon core [1, 5, 12, 16]. These observations point towards a multitude of modes Phe can use when interacting with membranes. An account of possible Phe interaction modes is currently available in the literature [1, 5, 6, 9–13, 15–17]. However, what remains to be further investigated is precise molecular level details of the initial Phe/membrane interaction, e.g. Phe intercalation parameters, the effect of hydration, counterions, etc., and how they may be predetermining the cascade of events to follow. Such knowledge is crucial for the advancement of our understanding of disease mechanisms and for the development of new approaches to the design of therapeutic agents.

In this study, we used Langmuir monolayers made up of a single phospholipid as the simplest membrane models. The initial interaction of membranes with Phe was modeled by injecting Phe into the aqueous phase underneath the monolayer. The advantage of monolayers as membrane models is that they offer precise control over the lateral pressure (surface pressure, in monolayer terms) and area per phospholipid molecule. This provides a unique opportunity to measure the expansion of membrane area induced by a biomolecule dissolved in the aqueous phase, e.g. Phe, in the constant pressure insertion assay [18–20]. Area expansion data can then be used to estimate biomolecule intercalation parameters [18, 19]. In particular, in our study, area expansion data afforded determining the Phe intercalation area, *A*_Phe_. Augmented with epifluorescence microscopy, the assay also allowed to identify the membrane environment involved with Phe intercalation. Based on *A*_Phe_ values, Phe intercalation modes were assessed. Two modes have been found to operate in model membranes, one of them plausibly involving a counterion.

## Methods

### Materials

Both phospholipids, 1,2-dipalmitoyl-*sn*-glycero-3-phosphoethanolamine (DPPE) and 1,2-dipalmitoyl-*sn*-glycero-3-phosphocholine (DPPC), and the fluorescent probe, 1,2-dioleoyl-*sn*-glycero-3-phosphoethanolamine-N-(Lissamine Rhodamine B Sulfonyl) labeled at the headgroup (DOPE-Rh), were purchased from Avanti Polar Lipids, Inc. The phospholipids were > 99% pure and used without further purification. Phenylalanine (99%) and phosphate buffered saline (PBS) were purchased from Sigma. Solvents used were of HPLC grade from Fisher Scientific. Deionized water with 18.2 MΩ•cm resistivity was produced by a Milli-Q Synthesis water purification system (EMD Millipore, MA).

### Model membranes

Model membranes were prepared by spreading phospholipids, DPPE or DPPC, from chloroform solutions at the air/water interface in a KSV2000SP Langmuir trough (KSV Instruments Ltd., Finland). For spreading solutions, a phospholipid concentration of ~ 1 mg/mL was used. Spreading volume was adjusted such that model membranes were formed over the most of the trough area to make available as much of membrane surface as possible for interactions with Phe. To form model membranes, phospholipids were compressed to a packing density similar to that in a biological membrane. While the mean molecular area, *A*, reduced upon compression, the surface pressure, *π*, was recorded as *π* – *A* isotherms. A filter paper Wilhelmy plate was used to measure *π* to an accuracy of 0.1 mN/m. The trough was thermostated to maintain a physiologically relevant temperature of 37 ± 1 °C [21].

### Area expansion measurements

To study Phe-induced expansion of model membranes, the constant pressure insertion assay was used [18–20]. In this assay, monolayers were compressed to an area of ~ 52 and ~ 65 Å^2^/molecule for DPPE and DPPC, respectively, which corresponds to their typical packing densities in a biological membrane [22]. The monolayer pressure, *π*, was then set to maintain constant by the trough electronic feedback device controlling the movement of two barriers whereas the area per phospholipid molecule, *A*, was allowed to change. After 20 min to allow for a monolayer to equilibrate, Phe was injected underneath the monolayer and let to interact for 2 h. The Phe-induced area expansion was recorded over time, *t*, as *A* − *t* isotherms.

### Epifluorescence microscopy

For the EFM imaging of model membranes, the Langmuir trough was interfaced with an upright Nikon Eclipse FN1 epifluorescence microscope. To enable the imaging, phospholipid spreading solutions were labeled with ~ 0.5 mol% of fluorescent probe, DOPE-Rh. Imaging was performed with the Nikon TRITC HYQ filter combination (545CWL excitation filter, 570LP dichroic mirror and 620 CWL barrier filter) through a 10x objective. The images were captured by a CCD camera directly onto a computer screen. Image processing and analysis was performed with the Simple PCI 6 software (Compix Inc., PA).

## Results

Phe is known to have a propensity for partitioning in the hydrocarbon core of phospholipid membranes [1, 2]. When injected underneath model membranes, Phe caused an expansion of monolayer area in the constant pressure insertion assay. However, the Phe-induced area expansion strongly depended on Phe concentration. At Phe concentrations below ~ 0.25 mM, no expansion of monolayer area was detected. Phe-induced area expansion became noticeable above ~ 1.25 mM of Phe (see Additional file 1: Figure 1S). This concentration-dependent expansion of monolayer area indicates that, beyond a certain concentration threshold, Phe molecules collectively intercalate into the model membrane and may perturb phospholipid packing. To examine the effect of Phe intercalation on model membranes, the data presented below were obtained at ~ 2.5 mM of Phe. It is worth noting that this concentration is well within pathologic Phe levels [5, 9, 10, 23].

### In-situ imaging of Phe intercalation

**Fig. 1:**
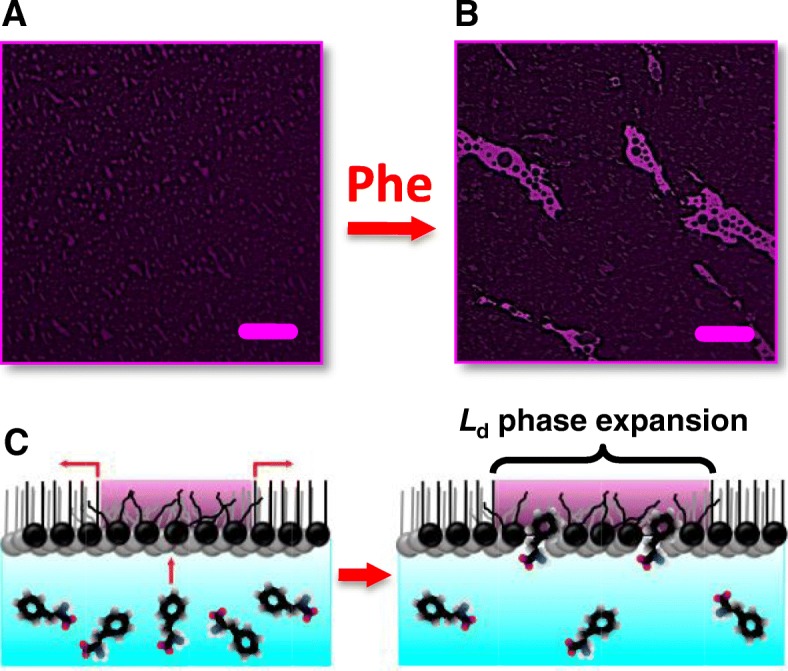
Phe intercalation into the DPPE model membrane. EFM images show the membrane morphology before (**a**) and 2 h after (**b**) Phe injection. Red staining indentifies the membrane *L*_d_ phase. Phe injection causes the area of *L*_d_ phase to expand plausibly due to the intercalation of Phe, which is depicted in the schematic (**c**). Images are for a DPPE monolayer initially at an *A* of ~ 52 Å^2^/molecule on PBS at 37 °C. Scale bar is 50 μm

**Fig. 2:**
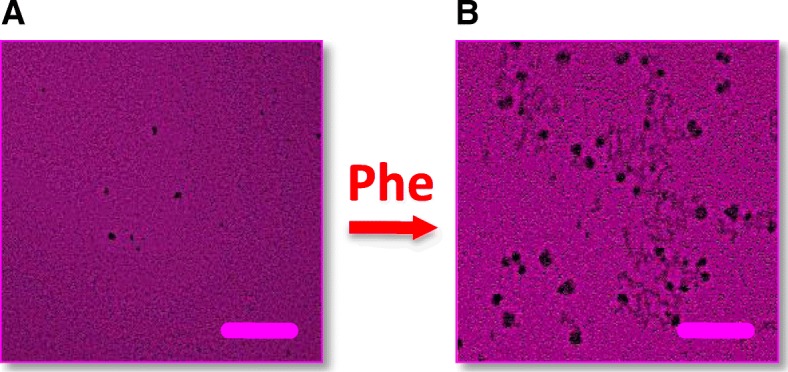
Phe intercalation into the DPPC model membrane. EFM images show the membrane morphology before (**a**) and 2 h after (**b**) Phe injection. Red staining indentifies the membrane *L*_d_ phase. Images are for a DPPC monolayer initially at an *A* of ~ 65 Å^2^/molecule on PBS at 37 °C. Scale bar is 50 μm

The effect of Phe intercalation on model membranes was first visualized with EFM. According to a recent study [5], Phe is likely to intercalate into the membrane through small-scale packing defects in the liquid-disordered (L_d_) phase. Hence, in this study, we used DOPE-Rh as the fluorescent probe to image the L_d_ phase. The probe is known to preferentially partition in this phase [24]. The L_d_ phase thus appears stained red in the images (in Figs. 1 and 2 discussed below).

Figure 1 shows images of a DPPE monolayer before and after Phe injection. The monolayer was compressed to an area of ~ 52 Å^2^/molecule and kept at a constant pressure. Dark field in image A captured before Phe injection indicates that, at 52 Å^2^/molecule, DPPE is predominantly in the gel state. The gel phase appears dark in EFM images because the fluorescent probe, DOPE-Rh, is excluded from it [24]. DPPE typically forms the *L*_β_ gel [25]. In the *L*_β_ gel, the hydrocarbon chains of DPPE are untilted as depicted in the schematic in Fig. 1c. Red fluorescent domains in the image suggest that the fluid L_d_ phase is also present (see the same image with a 1.5x magnification in Additional file 1: Figure 2S (image A) ). In the L_d_ phase, the hydrocarbon chains are disordered. The L_d_ phase is depicted stained red in the schematic in Fig. 1c.

The disorder of L_d_ phase allows dissolved biomolecules to interact with membranes, which makes this phase the most biologically relevant. Indeed, the comparison of images in Fig. 1 shows a noticeable expansion of red L_d_ phase domains 2 h after Phe injection, plausibly due to Phe intercalation. This is depicted in the schematic in Fig. 1c. Although the monolayer morphology remains essentially the same, the area fraction of fluorescent L_d_ phase domains in image B has increased by ~ 10% as compared to image A. Moreover, when expanding, some of the L_d_ domains appear to merge in trails propagating through the gel phase (image B in Fig. 1). Such L_d_ phase trails started to appear in DPPE monolayers ~ 1 h after Phe injection and were highly reproducible. These observations thus confirm that Phe intercalates into the model DPPE membrane primarily through the L_d_ phase. (To rule out any artifacts, control measurements were performed with DPPE alone. In these experiments, a DPPE monolayer was compressed to the same area of 52 Å^2^/molecule and kept for 2 h at a constant pressure without Phe injection. EFM imaging showed a contraction of red L_d_ phase domains over time (see Additional file 1: Figure 2S), which is completely opposite to the trend seen in Fig. 1b.)

As compared to DPPE, the effect of Phe injection on model DPPC membranes is more intricate. Figure 2 shows images of a DPPC monolayer before and after Phe injection. The monolayer was compressed to an area of ~ 65 Å^2^/molecule. The latter value, although larger than that for DPPE, corresponds to the typical DPPC packing density in a biological membrane [22]. As seen in EFM images in Fig. 2, Phe induces striking changes in the DPPC monolayer morphology. Tiny dark gel domains in a sea of red fluorescent fluid phase in image A indicate a two-phase coexistence in the DPPC monolayer before Phe injection. Starkly different from the signature kidney-shaped domains usually observed in DPPC monolayers at 20 °C, these tiny gel phase domains appear to predominate in the morphology of DPPC monolayers at elevated temperatures as has been visualized by other imaging techniques [26, 27]. Image B captured 2 h after Phe injection, reveals that both the number and size of the dark gel domains significantly increases. This points towards a massive nucleation and growth of the DPPC gel phase, which is likely triggered by Phe intercalation. A similar trend was reported for DPPC/Phe interactions at 20 °C [5]. Interestingly, the effect of Phe on DPPC monolayers appears to contrast with that observed for DPPE, which might imply a difference in Phe intercalation parameters. A series of measurement was thus performed to determine the intercalation area of Phe for both DPPC and DPPE membranes as discussed below.

### Phenylalanine-induced expansion of model membranes

**Fig. 3:**
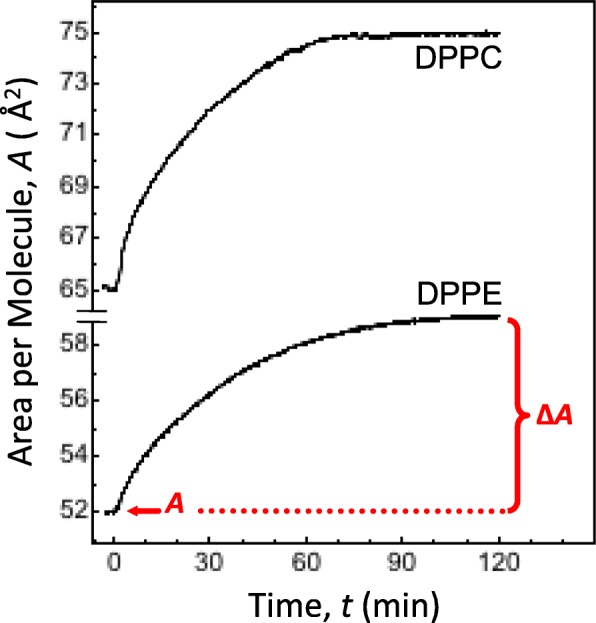
Typical area expansion *A* − *t* isotherms from insertion assays. The isotherms are for a DPPE and DPPC monolayer initially at an *A* of ~ 52 and ~ 65 Å^2^/molecule, respectively. Time *t* = 0 in the isotherms corresponds to Phe injection point. Δ*A* indicates the Phe-induced area expansion in the steady state. By relating Δ*A* to *A*, the relative area expansion, Δ*A*/*A*, is obtained. The data are for monolayers on PBS at 37 °C

The Phe-induced expansion of membrane area was also measured with the Langmuir balance. The measurements were performed simultaneously with EFM imaging by using the constant pressure insertion assay protocol [18–20]. A second set of area expansion measurements without EFM was performed as well to avoid labeling the model membranes with DOPE-Rh. These measurements were designed as a control series to assess the effect of fluorescent probe, if any, on Phe/membrane interactions. Both series of measurements produced identical results thus indicating that the use of DOPE-Rh did not give rise to any artifacts. Figure 3 shows typical *A* − *t* isotherms from the insertion assays. As seen in the figure, Phe injection caused the area per phospholipid molecule, *A*, to increase over time. For both monolayers, *A* − *t* isotherms leveled off to a steady-state value of *A* ~ 1.5 h after Phe injection as shown in Fig. 3.

The DPPE monolayer, initially at 52 Å^2^/molecule, exhibits a ~ 7 Å^2^ increase in *A* when its interactions with Phe reach the steady state. This Phe-induced area expansion is denoted as Δ*A* in Fig. 3. By relating Δ*A* to the initial area, *A* (before Phe injection), the relative area expansion, Δ*A*/*A*, can be estimated. For DPPE, Δ*A*/*A* expressed in % amounts to ~ 12%. The latter value correlates well with the ~ 10% increase of the area fraction of fluorescent L_d_ phase observed in the EFM images in Fig. 1. For the DPPC monolayer, somewhat larger Δ*A* values were recorded. When related to the initial DPPC area of 65 Å^2^/molecule, they yielded a value of ~ 15% for Δ*A*/*A*. The *A* − *t* data thus confirm that both model membranes undergo a noticeable area expansion upon Phe injection. This suggests that Phe is capable of intercalating both DPPE and DPPC packing.

### Phenylalanine intercalation area

A number of studies have demonstrated that the relative area increase, Δ*A*/*A*, can be used to calculate the area a biomolecule takes when inserting in a model membrane [18, 19]. Based on the simple Boltzmann factor [18], the relationship between Δ*A*/*A* and Phe intercalation area, *A*_Phe_, can be written as.

ln (Δ*A*/*A*) ≈ − *πA*_Phe_/*kT* (1).

Here, *π* is the surface pressure, *k* is the Boltzmann constant and *T* is the temperature [18]. As follows from eq. 1, a semilogarithmic plot of Δ*A*/*A* as a function of *π* should yield a regression line with a negative slope − *A*_Phe_/*kT*. From the slope of the line, a value for *A*_Phe_ can then be determined.

**Fig. 4:**
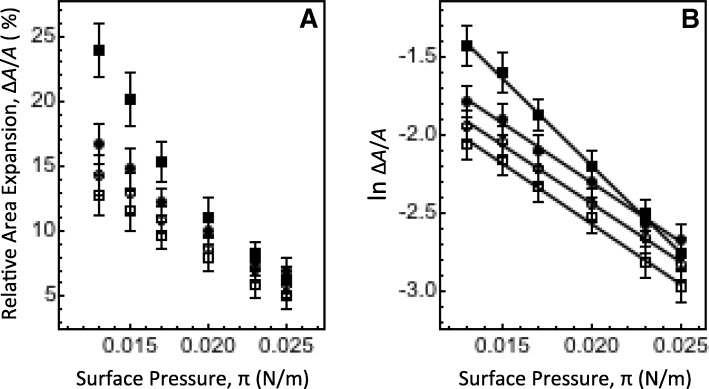
Phe insertion assay data analysis. **a** Steady-state Δ*A*/*A* as a function of *π* for DPPE and DPPC monolayers on water (open circles and squares, respectively) and PBS (filled circles and squares, respectively). **b** Steady-state Δ*A*/*A* as a function of *π* on a semilogarithmic scale. Solid lines are linear fit to the data. From the slope of linear fits, two different values for *A*_Phe_ were obtained. For the DPPC monolayer on PBS (filled squares, slope = − 112 ± 7), a value of 48 ± 3 Å^2^ was found for *A*_Phe_ with eq.1. For other monolayers, a value of 33 ± 2 Å^2^ was found for *A*_Phe_ from the virtually identical slopes of − 77 ± 2, − 76 ± 3 and − 78 ± 2. The data are for DPPE and DPPC monolayers initially at 52 ± 4 and 65 ± 5 Å^2^/molecule, respectively

To enable the use of eq. 1, a set of Δ*A*/*A* values was obtained over a wider range of *A* centered at 52 and 65 Å^2^/molecule for DPPE and DPPC monolayer, respectively. For DPPE, Δ*A*/*A* values were measured for monolayers with the initial area in a range of 48–56 Å^2^/molecule. For DPPC, the measurements were performed over a range of 60–70 Å^2^/molecule. For the measurements, the constant pressure insertion assay protocol was used. Values of *π* corresponding to the selected *A* ranges span from ~ 13 to 25 mN/m (For correlation between *π* and *A* values, see Additional file 1: Figure 3S ). Figure 4 shows the Δ*A*/*A* data for both monolayers as a function of *π*. As seen in Fig. 4a, Phe caused noticeable expansion in both monolayers over the entire range of selected *A* and *π* values. Somewhat larger relative area expansion was observed for DPPC monolayers on PBS. For comparison, Fig. 4 also displays the Δ*A*/*A* data for both monolayers on water, which is discussed in more detail in the section 3.4 below. All monolayers showed a decrease in the Phe-induced Δ*A*/*A* with increasing *π* (Fig. 4a). Phe ceased to cause the expansion of monolayers area at a *π* of ~ 32 mN/m (see Additional file 1: Figure 4S).

To determine the Phe intercalation area, *A*_Phe_, the Δ*A*/*A* data are plotted on a semilogarithmic scale as a function of *π* in Fig. 4b. As seen in the figure, all datasets produce linear regressions with a negative slope as predicted by eq. 1. This indicates that Phe intercalates in the model membranes with a constant *A*_Phe_ [18, 19]. However, there is a marked difference in the regression slope between the dataset for DPPC on PBS and other monolayers. Based on eq. 1, this suggests two different *A*_Phe_ values. Indeed, from the slopes of linear fits in Fig. 4b, the intercalation areas of 33 ± 2 and 48 ± 3 Å^2^ were obtained for Phe. The former value correlates with the area of ~ 28 Å^2^ estimated for a molecule of aromatic amines [17], whereas the latter value points towards a larger Phe species (e.g. a sodiated Phe complex [28, 29]) intercalating in the DPPC monolayer from PBS as discussed below.

## Discussion

### Counterion in Phe intercalation

**Fig. 5:**
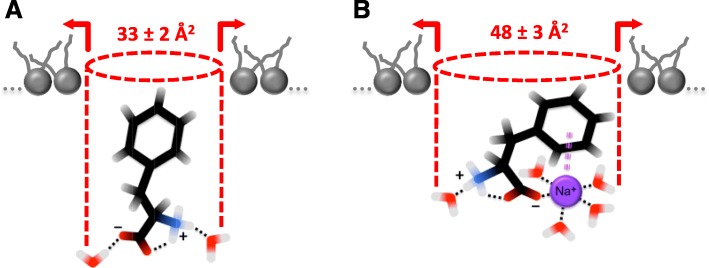
Two modes of Phe intercalation into model membranes. **a** The intercalation mode involving a Phe monomer. In this mode, the phenyl ring inserts between phospholipid molecules in the *L*_d_ phase nearly normal to the membrane surface, which is consistent with the *A*_Phe_ value of 33 ± 2 Å^2^. Phe may retain its hydration water molecules as shown with dotted traces. **b** The intercalation mode involving a *π* − cation complex. In the complex, a cation, e.g. Na^+^, links the Phe ring and carboxylate group through its first coordination shell [25], which is shown with dotted traces. This is likely to confine the Phe ring in a titlted conformation to the membrane headgroup region, thereby taking up a larger area of 48 ± 3 Å^2^ as indicated with dashed lines

Among the aromatic amino acids, Phe exhibits the largest conformational variety [30–32]. Phe conformers mainly differ in the degree of alanyl chain folding with respect to the Phe ring [30]. This conformational variety might allow Phe to use different modes when binding to surfaces and interfaces. In particular, Phe is often viewed as stretched along the surface normal with the Phe ring embedded in the nonpolar phase and its charged groups facing the polar phase [4, 5, 15–17, 33]. Based on the data available in the literature, the area per Phe molecule in such a conformation ranges from 24 to 33 Å^2^ [4, 17, 33]. The latter correlates well with the *A*_Phe_ value of 33 ± 2 Å^2^ obtained in our study. This value is thus consistent with a Phe monomer intercalating into the model membranes by inserting the phenyl ring into the membrane hydrocarbon core nearly normal to the membrane surface with its headgroup positioned in the aqueous region of the interface as predicted by earlier studies [5, 15]. Figure 5a schematically depicts this mode of Phe intercalation. Found at the higher range of reported Phe areas, the value of 33 ± 2 Å^2^ may also suggest that the headgroup of Phe remains at least partially hydrated. In fact, hydration of Phe with two water molecules, ammonium- and carboxyl-bound, has been shown to present a stabilizing structural element in the amino acid molecule [34]. The two water dipoles line up favorably with the charged groups and help maintaining the relative positions of the phenyl ring and alanyl chain in the molecule [34]. However, this mode appears to be valid primarily for Phe intercalation into model membranes formed on water, that is when no counterions are present. The difference in the *A*_Phe_ values obtained for monolayers on PBS suggests that the addition of saline may significantly affect the mode of Phe intercalation, in particular for DPPC.

The rather large value of *A*_Phe_ = 48 ± 3 Å^2^ obtained for the DPPC monolayer on PBS points towards the intercalation of Phe either in a different conformation or as a larger species. In aqueous solutions, aromatic *π* − rings have been shown to successfully compete with water in binding to alkali metal cations and stably retain them in a *π* − cation complex structure [28, 29]. In particular, Na^+^ cation (present in PBS at a concentration of ~ 130 mM) has been suggested to simultaneously interact with the *π-*electron cloud and carboxylate group of zwitterionic Phe [28, 29]. In this Na^+^(Phe) complex structure, Na^+^ cation sheds two water molecules of its first coordination shell to link the phenyl ring and carboxylate group via a bidentate geometry [29]. This is drawn schematically in Fig. 5b. Such a complexation with Na^+^ is likely to anchor the Phe ring to the aqueous phase and encourage the folding of alanyl chain. Instead of stretching into the hydrocarbon region, the sodiated Phe ring may thus adopt a tilted conformation in the headgroup region, thereby taking up a larger area when inserting in the model membrane. In fact, a study by Yang et al. [33] suggests that mere tilting of Phe ring with respect to the surface normal causes the area occupied by the amino acid molecule to increase. Moreover, Na^+^ will also add to the area of sodiated Phe complex. A value of ~ 2.3 Å has been reported for the radius of hydrated Na^+^ cation [35], which corresponds to an area of ~ 16.6 Å^2^. The latter appears to correlate with the ~ 15 Å^2^ increase in *A*_Phe_ (from 33 ± 2 Å^2^ on water to 48 ± 3 Å^2^ on PBS) observed for DPPC in our study, which is consistent with Phe dragging along Na^+^ and intercalating into the membrane as a Na^+^(Phe) complex.

### Biological implications of Phe intercalation

The difference between the *A*_Phe_ values obtained for DPPE and DPPC model membranes in our study suggests that Phe is indeed capable of using at least two distinct modes of intercalation. This may have implications for a number of processes triggered by elevated extracellular Phe levels. In particular, the intercalation of Phe involving a counterion, e.g. Na^+^ depicted in Fig. 5b, may have a role in the accumulation of amyloid-like Phe deposits at the membrane surface under pathological conditions. Of the most abundant membrane phospholipids, DPPC is located primarily in the outer leaflet of the membrane exposed to the extracellular fluid [36]. Phe intercalation, which is feasible at elevated Phe levels [5, 9–12], is thus likely to use the mode involving a counterion as depicted in Fig. 5b for the DPPC model membrane. Elevation in extracellular Phe can also induce the self-assembly of Phe into amyloid fibrils [9, 10]. This self-assembly is believed to occur through the parallel *π*–stacking of Phe rings [9, 11, 13]. Although counterions have been shown to drive the Phe stacking by stabilizing the self-assembled amyloid structures [13], the exact mechanism of interactions involved with the deposition of Phe fibrils onto the membrane surface remains largely unclear. The findings of this study enable us to hypothesize that the intercalation of Phe in a titled configuration exposing the sodiated Phe ring may in fact provide nucleation and/or docking sites for the self-assembly and anchoring of Phe amyloid fibrils at the membrane surface.

## Conclusions

Phe is capable of intercalating through the *L*_d_ phase into both DPPE and DPPC model membranes. Two modes of intercalation are likely to be involved. The modes differ by the *A*_Phe_ area taken by each Phe molecule upon intercalation. The area of *A*_Phe_ 33 ± 2 Å^2^ is consistent with a Phe monomer intercalating into membrane by inserting the phenyl ring nearly normal to the membrane surface. This mode operates in DPPE membranes in both absence and presence of counterions. For DPPC membranes however, counterions appear to alter the mode of Phe intercalation. The value of *A*_Phe_ = 48 ± 3 Å^2^ suggests that, from saline, Phe tends to intercalate into DPPC membranes as a larger species, e.g. a Na^+^(Phe) complex. Complexation with Na^+^ is likely to anchor Phe ring to the aqueous phase, which may provide nucleation sites for Phe self-assembly. These findings can be instrumental in understanding various membrane processes triggered by interactions with Phe, in particular the early events leading to the deposition of Phe amyloid fibrils onto the membrane surface.

## Additional file

Additional file 1: Additional data for this article including Phe intercalation isotherms, EFM images of DPPE monolayers and *π* − *A* isotherms for DPPE and DPPC monolayers can be found in the Supplementary Information file. **Figure 1S.** Steady-state Δ*A* as a function of *C*_Phe_ for DPPE (filled circles) and DPPC (filled squares) monolayers on PBS at 37 ± 1 °C. A set of three *A*−*t* isotherms was analyzed for each data point. Error bars indicate the standard deviation within each set of three repeat measurements. **Figure 2S.** EFM images of a DPPE monolayer held at a constant pressure for 20 min (A) and 2 h (B). Red staining indentifies the membrane *L*_d_ phase. Images are for a monolayer compressed to an *A* of 52 Å^2^/molecule on PBS at 37 ± 1 °C. A 1.5x magnification was applied to images to make features more visible. Scale bar is 50 μm.. **Figure 3S.** *π*−*A* Isotherms for DPPE and DPPC monolayers on PBS at 37 ± 1 °C. **Figure 4S.** Steady-state Δ*A* as a function of *π* for DPPE (filled circles) and DPPC (filled squares) monolayers on PBS at 37 ± 1 °C. A set of at least three *A*−*t* isotherms was analyzed for each data point. Error bars indicate the standard deviation within each set of three repeat measurements. (DOCX 367 kb)

## Abbreviations

DOPE-Rh: 1,2-dioleoyl-*sn*-glycero-3-phosphoethanolamine-N-(Lissamine Rhodamine B Sulfonyl)
DPPC: 1,2-dipalmitoyl-*sn*-glycero-3-phosphocholine
DPPE: 1,2-dipalmitoyl-*sn*-glycero-3-phosphoethanolamine
EFM: epifluorescence microscopy
PBS: phosphate buffered saline
Phe: Phenylalanine
