# Supplementary material for: Phenylalanine intercalation parameters for liquid-disordered phase domains – a membrane model study
Source: BMC Biophys. 2018 Nov 15;11:6. doi: 10.1186/s13628-018-0047-z (PMC6237005; doi:10.1186/s13628-018-0047-z)
Supplement: Supplementary file 1 — Additional data for this article including Phe intercalation isotherms, EFM images of DPPE monolayers and π − A isotherms for DPPE and DPPC monolayers can be found in the Supplementary Information file. Figure 1S. Steady-state ΔA as a function of CPhe for DPPE (filled circles) and DPPC (filled squares) monolayers on PBS at 37 ± 1 °C. A set of three A−t isotherms was analyzed for each data point. Error bars indicate the standard deviation within each set of three repeat measurements. Figure 2S. EFM images of a DPPE monolayer held at a constant pressure for 20 min (A) and 2 h (B). Red staining indentifies the membrane Ld phase. Images are for a monolayer compressed to an A of 52 Å2/molecule on PBS at 37 ± 1 °C. A 1.5x magnification was applied to images to make features more visible. Scale bar is 50 μm.. Figure 3S. π−A Isotherms for DPPE and DPPC monolayers on PBS at 37 ± 1 °C. Figure 4S. Steady-state ΔA as a function of π for DPPE (filled circles) and DPPC (filled squares) monolayers on PBS at 37 ± 1 °C. A set of at least three A−t isotherms was analyzed for each data point. Error bars indicate the standard deviation within each set of three repeat measurements. (DOCX 367 kb) [file 13628_2018_47_MOESM1_ESM.docx]

**Supplementary Information**

**Adamczewski et al.**

**Phenylalanine Intercalation Parameters for Liquid-Disordered Phase Domains – a Membrane Model Study**

**Phe Intercalation Isotherms.** Analysis of the data available in the literature suggests that Phe intercalation capacity is likely to be coupled to its concentration, *C*_Phe_, in the aqueous phase. Hence, a series of experiments was performed to determine the threshold value of *C*_Phe_ for Phe intercalation. The experiments were performed with DPPE and DPPC monolayers initially at ~52 and ~65 Å^2^/molecule, respectively. By gradually increasing the volume of Phe injection into PBS underneath the monolayers, *C*_Phe_ was systematically varied from 0.25 to 5 mM. A set of *A*−*t* isotherms was collected to assess the Phe-induced expansion of monolayer area, Δ*A* (see Figure 3 and related discussion in the main text), over the *C*_Phe_ range. The data are plotted as a function of *C*_Phe_ in Figure 1S.


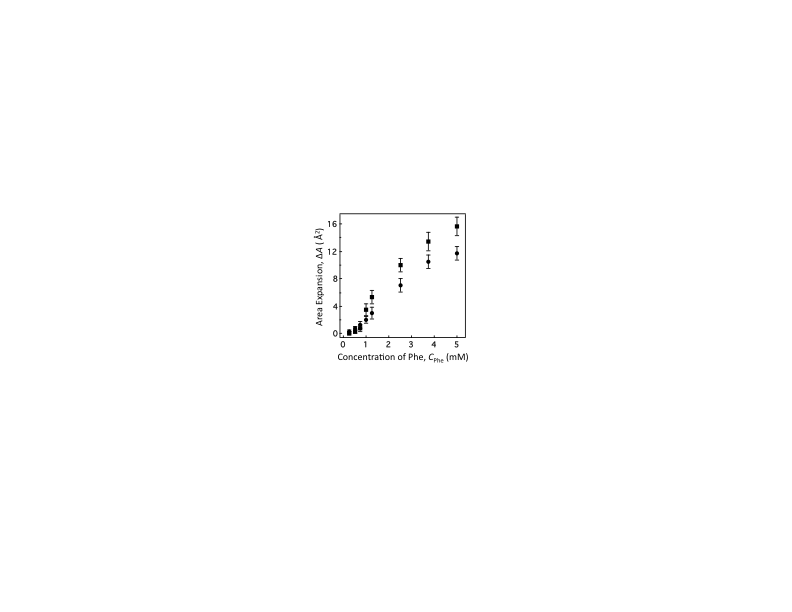


**Figure 1S.** Steady-state Δ*A* as a function of *C*_Phe_ for DPPE (filled circles) and DPPC (filled squares) monolayers on PBS at 37 ± 1 °C. A set of three *A*−*t* isotherms was analyzed for each data point. Error bars indicate the standard deviation within each set of three repeat measurements.

**
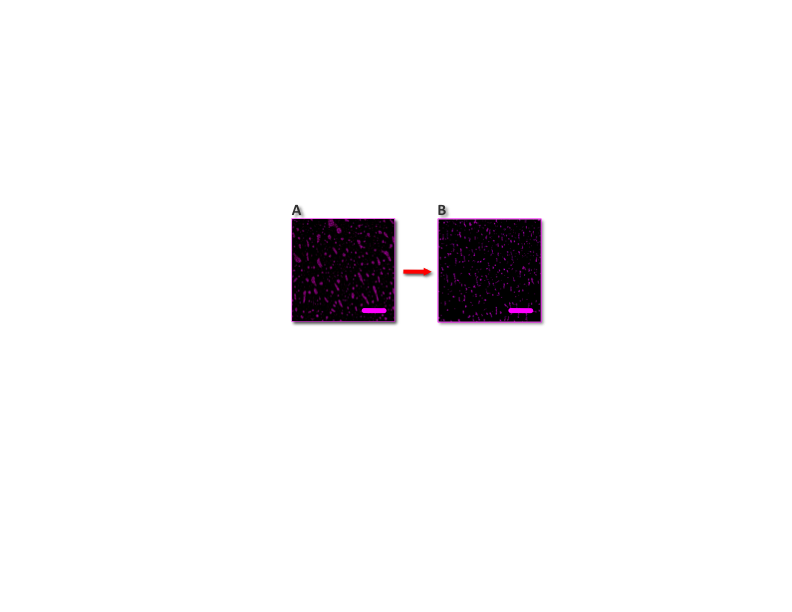
EFM imaging of DPPE monolayers.** A control series of EFM measurements was performed for DPPE monolayers to track changes in their morphology over time. Figure 2S displays images of a DPPE monolayer compressed to an area of 52 Å^2^/molecule and then kept at a constant pressure without Phe injection. As seen in the images, a noticeable contraction of red L_d_ phase domains occurred over time.

**Figure 2S.** EFM images of a DPPE monolayer held at a constant pressure for 20 min (A) and 2 h (B). Red staining indentifies the membrane *L*_d_ phase. Images are for a monolayer compressed to an *A* of 52 Å^2^/molecule on PBS at 37 ± 1 °C. A 1.5x magnification was applied to images to make features more visible. Scale bar is 50 μm.

***
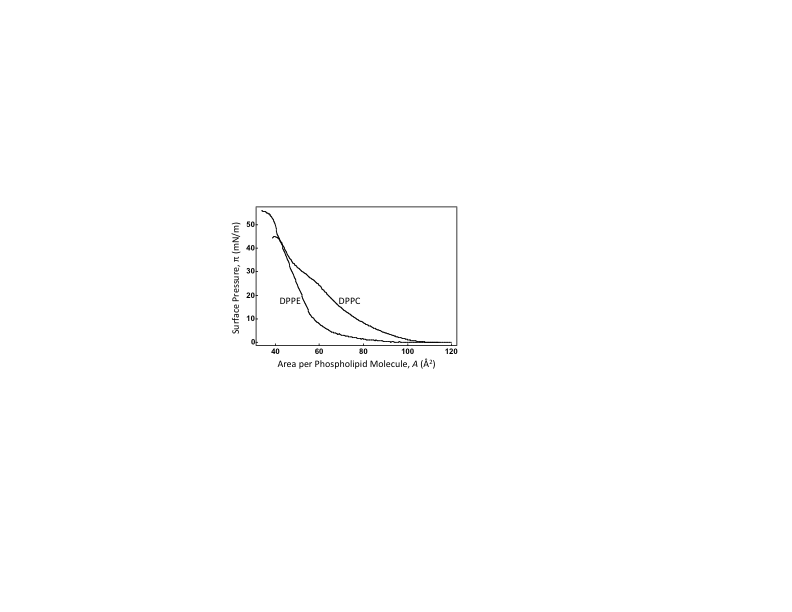
π*−*A* Isotherms for DPPE and DPPC monolayers.** The isotherms were measured for both monolayers and used to derive *π* values corresponding to the typical phospholipid packing densities in a biological membrane. The isotherms are shown in Figure 3S.

**Figure 3S.** *π*−*A* Isotherms for DPPE and DPPC monolayers on PBS at 37 ± 1 °C.

**
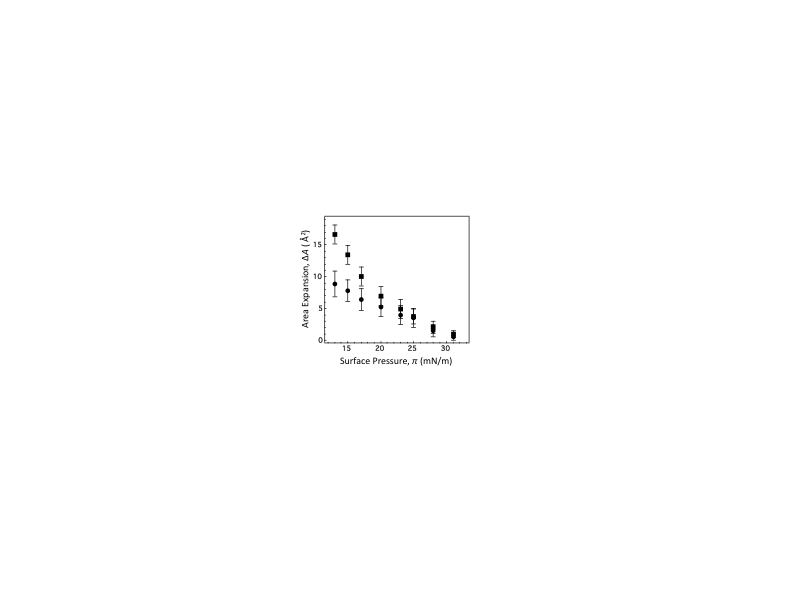
Intercalation capacity of Phe for model membranes.** The intercalation capacity of Phe for model membranes was examined over a range of *π* from ~13 to 35 mN/m with a ~2 mN/m increment. A series of constant pressure insertion assays were performed to obtain Δ*A* values corresponding to different membrane pressures in the range. The Δ*A* values are plotted as a function of *π* in Figure 4S. As follows from the plots in Figure 4S, Phe is capable of causing the area expansion and intercalating into membranes with *π* < 32 mN/m. The latter is within the estimated range of lateral pressures in biological membranes [18,20,21].

**Figure 4S.** Steady-state Δ*A* as a function of *π* for DPPE (filled circles) and DPPC (filled squares) monolayers on PBS at 37 ± 1 °C. A set of at least three *A*−*t* isotherms was analyzed for each data point. Error bars indicate the standard deviation within each set of three repeat measurements.
